# Bacterial Granulomatous Lung Diseases: Radiological Findings and Differential Diagnosis

**DOI:** 10.3390/idr18030053

**Published:** 2026-05-28

**Authors:** Stefano Giusto Picchi, Augusto Minieri, Francesco Lassandro, Giuseppe Russo, Giulia Lassandro

**Affiliations:** 1 Department of Radiology, Ospedale Pellegrini, ASL Napoli 1 Centro, 80134 Naples, Italy; stefanopicchi@libero.it; 2 Division of Radiology, UOC Diagnostica per Immagini e Radioterapia, University of Naples Federico II, 80138 Naples, Italy; augusto.minieri94@gmail.com; 3 Department of Radiology, Ospedale S. Anna e SS. Madonna della Neve, ASL NA3 Sud, Via Lenze, Boscotrecase, 80042 Naples, Italy; f.lassandro@gmail.com; 4 General Direction for Health Management, ASL Napoli 3 Sud, Via Marconi, Torre del Greco, 80059 Naples, Italy

**Keywords:** granulomatous lung disease (GLD), high-resolution computed tomography (HRCT), pneumonia, bacteria, *Mycobacterium tuberculosis*, tuberculosis (TB), *Mycobacterium avium* complex, *Brucella*, Q-fever, cat scratch disease

## Abstract

Background Granulomatous lung diseases include a spectrum of disorders, both infectious and noninfectious, unified by the presence of granulomas in the lung parenchyma. Granulomas are microscopic, organized collections of immune cells that arise as a response to persistent antigenic stimulation. Infectious granulomatous lung diseases arise from a variety of microbial agents, that include most frequently *Mycobacterium tuberculosis*, non-tuberculous mycobacteria, *Nocardia*, and *Borrelia*, as well as a wide range of fungal pathogens including *Histoplasma*, *Cryptococcus*, *Pneumocystis,* and *Aspergillus* species. Methods and Results: Definitive diagnosis is achieved through direct identification and subsequent culture of the causative pathogen in appropriate clinical specimens, including sputum, bronchoscopic samples, gastric aspirates, or pleural fluid. Imaging is fundamental for the detection and characterization of pulmonary granulomas. HRCT allows precise assessment of the number, size, and distribution of granulomatous lesions, can suggest an infectious etiology based on specific imaging patterns, and is essential for monitoring response to therapy over time. Differential diagnosis is challenging due to the numerous different imaging appearances with whom granulomatous lung diseases may manifest. Conclusions: The purpose of our review is to describe the spectrum of infectious granulomatous lung diseases caused by bacterial pathogens, highlighting their diverse radiologic presentations in order to assist radiologists in recognizing these entities and improving diagnostic accuracy.

## 1. Introduction

Granulomatous lung diseases (GLDs) represent a diverse spectrum of pulmonary disorders, both infectious and noninfectious, unified by the presence of granulomatous inflammation within lung tissue. Granulomas are microscopic, organized collections of immune cells that arise as a response to persistent antigenic stimulation [1]. From a histopathological perspective, granulomas are composed of epithelioid macrophages, multinucleated Langhans-type giant cells, and surrounding lymphocytes, forming dense, focal structures that reflect a chronic inflammatory process.

The noninfectious GLDs encompass a wide range of disorders, including systemic inflammatory diseases such as sarcoidosis, reactions to environmental and occupational exposures such as talc inhalation and berylliosis, or vasculitic disorders such as granulomatosis with polyangiitis [2].

Infectious GLDs arise from a variety of microbial agents that include *Mycobacterium tuberculosis*, non-tuberculous mycobacteria, *Nocardia*, and *Borrelia*, as well as a wide range of fungal pathogens including *Histoplasma*, *Cryptococcus*, *Pneumocystis*, and *Aspergillus* species.

Histologically, granulomas can also be subdivided into necrotizing and non-necrotizing types. This distinction is clinically relevant because necrotizing granulomas are most often associated with infectious etiologies, reflecting tissue destruction driven by microbial proliferation and host immune response [3].

Caseation represents a form of coagulative-like necrosis that transforms the early exudative alveolar focus and the adjacent lung parenchyma into a dense, cheese-like material. During this process, the alveolar architecture collapses, yet elastic fibers from alveolar septa and blood vessels often remain embedded within the necrotic core, accounting for the firm and rubbery texture of many caseous nodules [4].

The diagnostic approach to GLDs relies on integrated evaluation, including clinical history, laboratory investigations, and imaging studies. Additional techniques, such as bronchoalveolar lavage (BAL), transbronchial needle aspiration, cryobiopsy, and video-assisted thoracoscopic surgery (VATS) biopsy can substantially improve diagnostic precision and guide etiologic classification [3].

In the setting of infectious GLDs, definitive diagnosis is achieved through direct identification and subsequent culture of the causative pathogen in appropriate clinical specimens, including sputum, bronchoscopic samples, gastric aspirates, or pleural fluid. This microbiological confirmation remains the gold standard for establishing the infectious nature of the disease. Serological testing may provide additional supportive information, particularly for certain fungal or atypical bacterial infections [1].

Imaging, and especially High-resolution Computed Tomography (HRCT), is fundamental for the detection and characterization of pulmonary granulomas. HRCT allows precise assessment of the number, size, and distribution of granulomatous lesions, can suggest an infectious etiology based on specific imaging patterns, and is essential for monitoring response to therapy over time. Contrast-enhanced computed tomography (CECT) and PET-CT further contribute by identifying metabolically active lesions and lymphoproliferative sites [1].

Despite the high diagnostic performance of HRCT, CECT, and PET-CT in detecting and following granulomatous lung lesions, accurate differential diagnosis remains challenging. This difficulty arises from the wide variety of underlying pathogenic mechanisms and the substantial overlap in imaging appearances among different GLDs.

The aim of this review is to describe the spectrum of infectious GLDs caused by bacterial pathogens, highlighting their diverse radiologic presentations in order to assist radiologists in recognizing these entities and improving diagnostic accuracy. Table 1 provides a summary of all the main findings and helps to outline the key differences between the GLDs described below (Table 1).

## 2. *Mycobacterium tuberculosis*

### 2.1. Epidemiology and Pathophysiology

Tuberculosis (TB), the infectious disease caused by the pathogen *Mycobacterium tuberculosis*, remains a major global health issue, especially in developing countries [5]. In 2023, it caused about 1.25 million deaths worldwide. TB likely returned as the leading cause of death from a single infectious agent and continues to be a top killer among immunocompromised patients.

Despite being easily treatable, treatment access remains limited, especially for multidrug-resistant types (at most 40%) [6].

A prompt diagnosis is crucial for public health and patient care, but unfortunately, TB bacilli are detectable in biological fluid in only a minority of cases, even in active pulmonary TB [7].

*M. tuberculosis* consists in an aerobic, non-motile bacillus that does not form spores. It is transmitted through airborne droplet nuclei expelled by individuals harboring the bacteria, primarily through coughing. The likelihood of contagion between individuals is influenced by the quantity of infectious droplets released, the duration of exposure, and its pathogenicity.

The highest risk of developing active TB is observed in immunocompromised or fragile patients, such as those suffering from malnutrition, children or the elderly, or those affected by malignancies.

TB starts when airborne bacilli are inhaled deep into the alveoli, where they are engulfed by resident alveolar macrophages. These macrophages interact with T-cells, leading to their transformation into epithelioid histiocytes [8]. These specialized macrophages, along with lymphocytes, organize into granulomas-structured cellular masses that contain but do not eradicate the infection, the so-called Ghon focus [9]. TB granulomas are typically characterized by a necrotic core with a caseous appearance, encircled by epithelioid macrophages and multinucleated giant cells.

As the disease progresses, the primary lesion may enlarge or, more frequently, heal, leaving a dense fibrotic scar, sometimes containing calcifications. In most cases, the initial infection is asymptomatic. Only 5% of infected individuals, typically immunocompromised patients, develop the progressive primary TB. However, TB remains latent both clinically and microbiologically for many years [5].

### 2.2. Clinical Findings

Patients with active pulmonary TB can be asymptomatic, but in some cases the active form may present with an acute febrile syndrome, non-productive cough, hemoptoe, pleuritic chest pain, dyspnea, night sweats, chills, and unintended weight loss.

When promptly diagnosed and adequately treated, TB patients generally become non-infectious within a short period and can achieve complete recovery [10].

### 2.3. Diagnosis

The definitive diagnosis requires isolation of *M. tuberculosis* through culture of a clinical specimen obtained from the patient [5].

**Figure 1:**
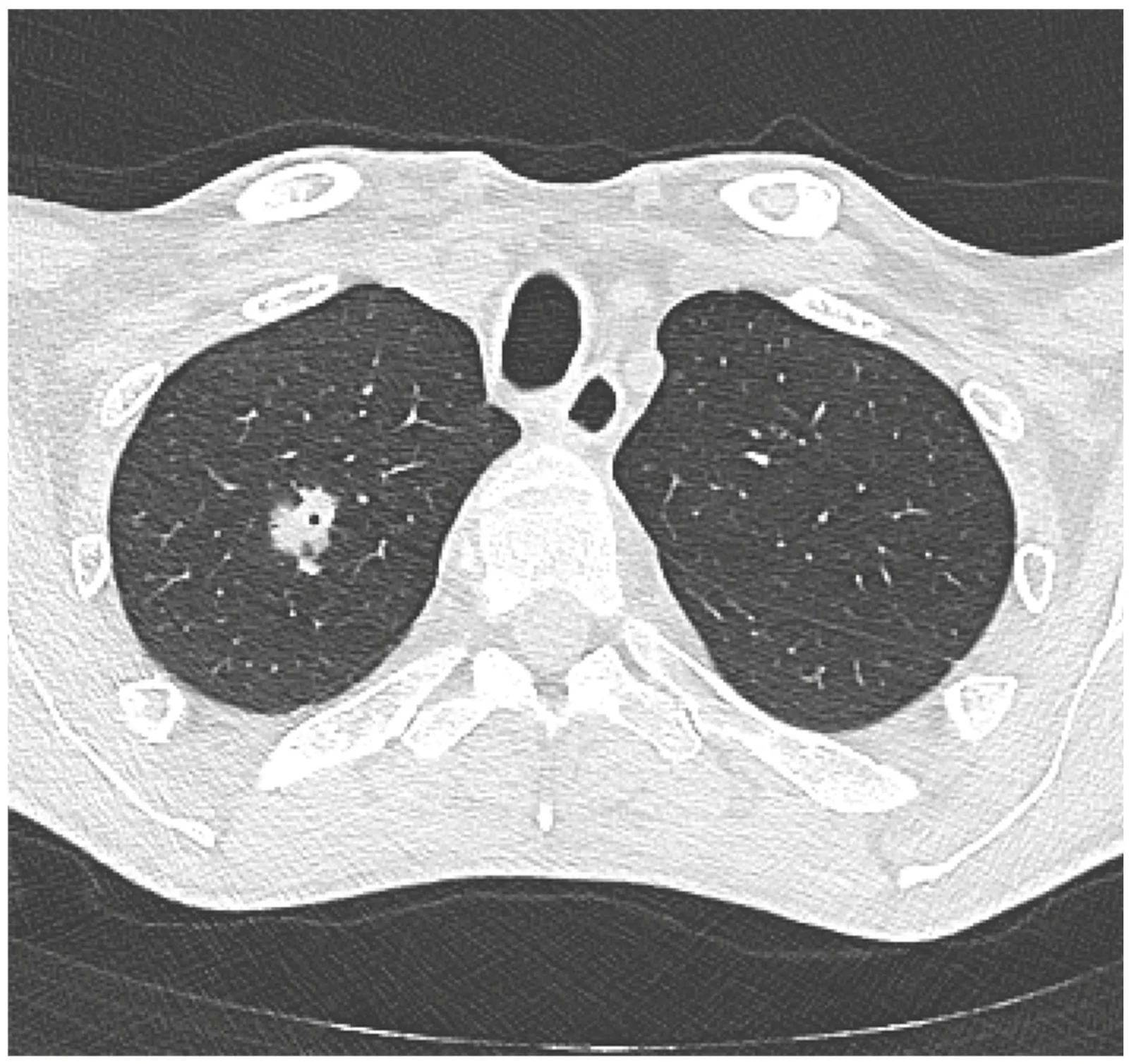
HRCT, axial plane. Primary pulmonary TB parenchymal lesion in the right upper lobe in an immunocompetent male patient. Initial cavitation is forming in the lesion.

In immunocompetent individuals, primary pulmonary TB typically begins with a localized parenchymal lesion that may either progress into an area of airspace consolidation or, more frequently, resolve through fibrotic transformation of the granulomatous tissue (Figure 1). In children, the most consistent feature is lymphadenopathy, most frequently unilateral hilar or paratracheal lymph node enlargement visible in chest X-ray [11]. In immunocompromised patients, atypical or uncommon radiologic manifestations of pulmonary TB are often observed. In this population, active pulmonary TB frequently manifest through multiple cavitary lesions that exhibit a non-segmental pattern [10,11].

When a calcified parenchymal lung lesion is associated with lymph nodes, it is called a Ranke complex [11].

Although primary TB is more prevalent in children, its occurrence in adults has been increasingly recognized [10]. As reported by Leung et al., the condition predominantly affects patients under five years of age.

There is ongoing debate regarding the most frequently affected pulmonary regions in primary TB. In fact, according to Koh et al., chest X-ray manifestations may include nodules, consolidation, or cavitation in the upper lobes, whereas mediastinal lymphadenopathy and pleural effusion are less common [12]. These imaging findings have traditionally been linked to post-primary or reactivation TB.

Parenchymal consolidation resulting from granulomatous inflammation is radiographically evident in approximately 70% of primary TB cases, typically in a unilateral distribution, without predilection for a specific lung zone. This consolidation may appear dense and homogeneous, although patchy, linear, nodular, or mass-like morphologies are also reported [12]. On CT, affected lymph nodes display central hypodensity, suggestive of caseous necrosis. Pleural effusions associated with primary TB are generally unilateral and ipsilateral to the parenchymal lesion. They may be sizable and occasionally present even in the absence of radiographically detectable pulmonary involvement.

In cases of reactivation TB, Koh et al. observed the presence of patchy consolidation and ill-defined nodules in chest X-rays, predominantly affecting the upper lobes. Cavitary lesions were noted only in a limited subset of patients [10,11,12].

**Figure 2:**
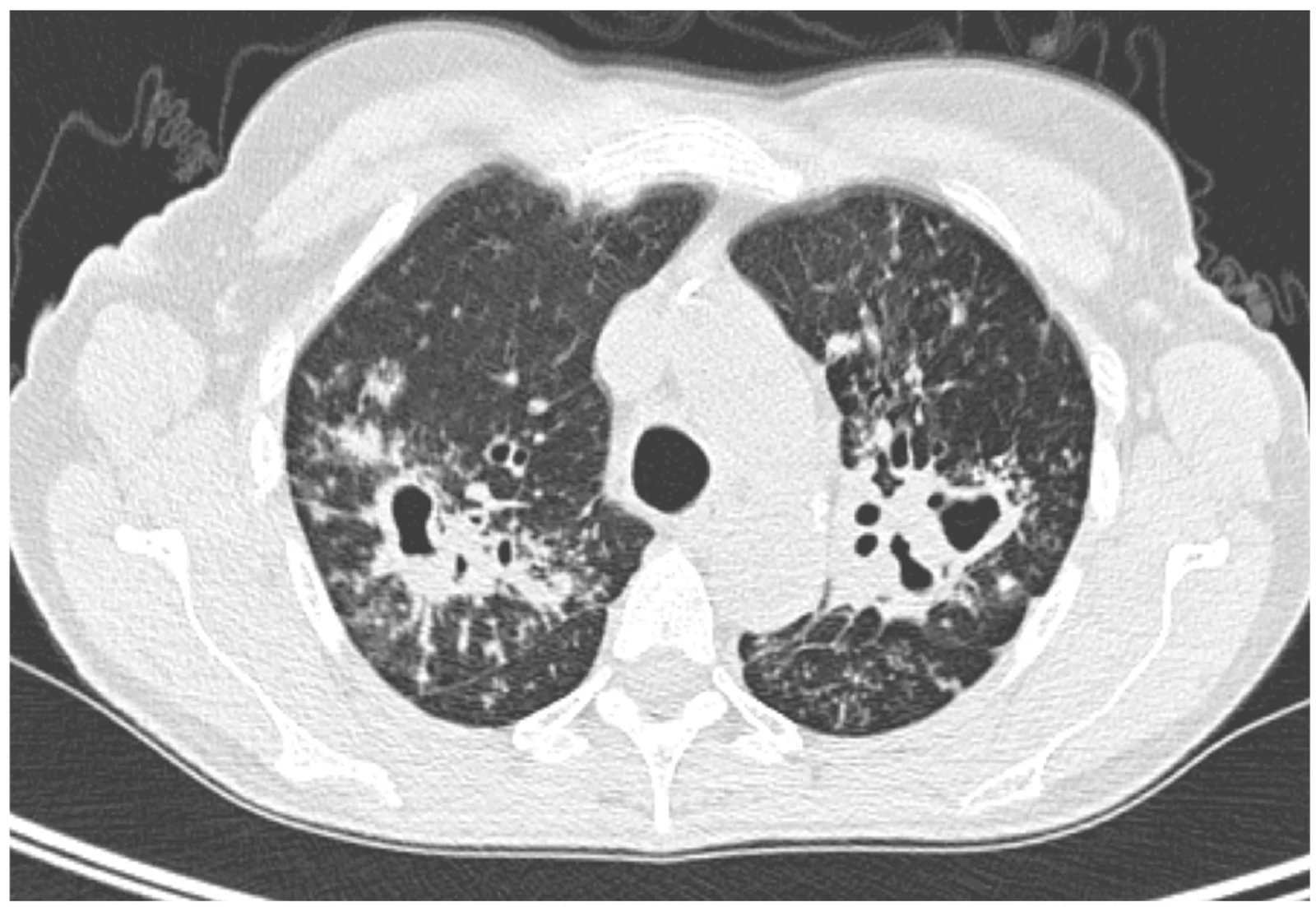
HRCT, axial plane. HRCT shows patchy heterogeneous consolidations involving posterior segments of the upper lobes, some of them cavitated, in a patient affected by reactivated TB.

**Figure 3:**
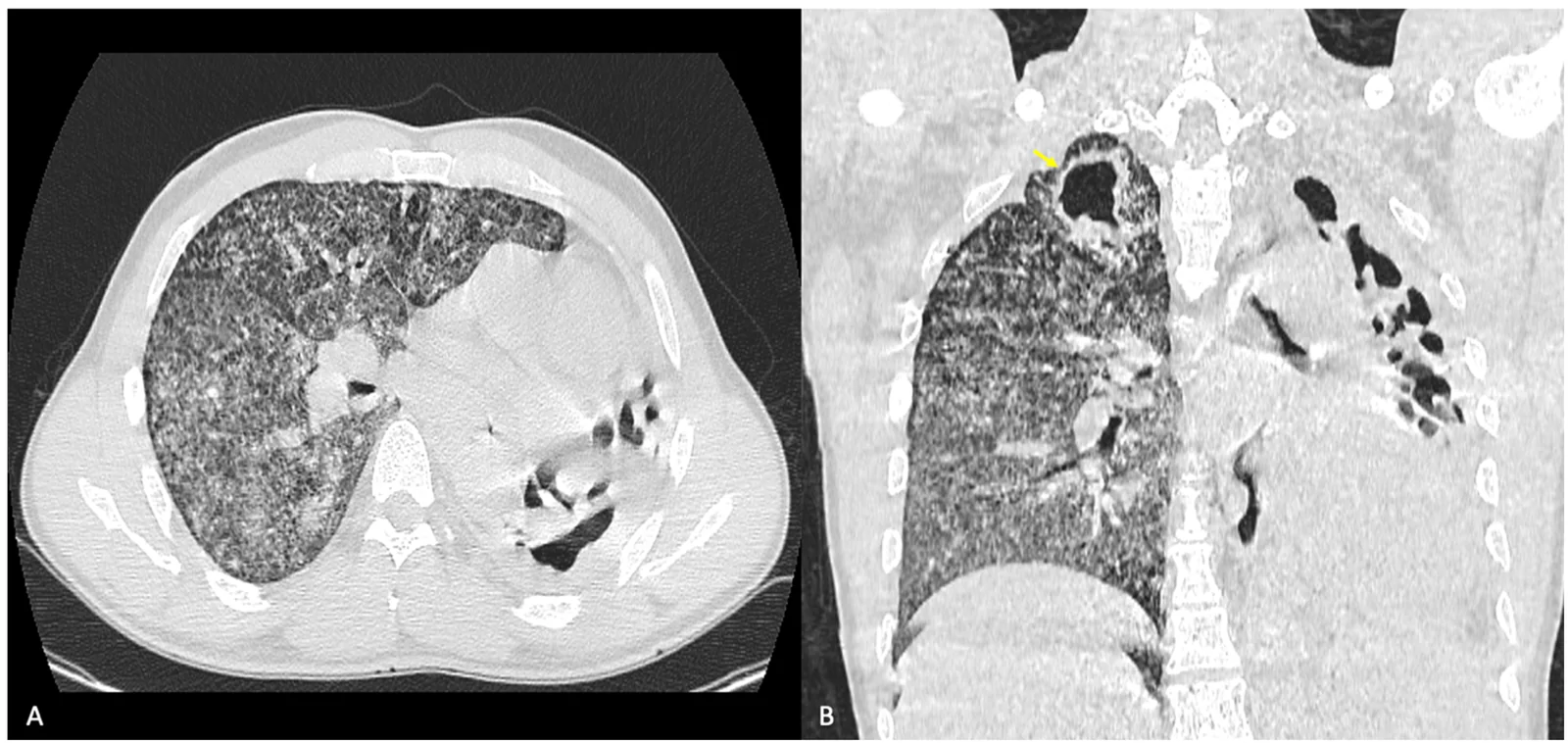
HRCT, axial (**A**) and coronal plane (**B**). A case of miliary TB presenting as countless 1–3 mm nodules randomly distributed in the entire right lung. In this case, other typical TB lung parenchymal abnormalities are present, as the extended cavitated pulmonary consolidations in the left lung and a tuberculoma in the upper right lobe ((**B**), yellow arrow).

CT commonly reveals focal or patchy heterogeneous consolidations involving preferentially the apical and posterior segments of the upper lobes, as well as the superior segments of the lower lobes (Figure 2). Poorly defined nodules and linear opacities are also typical findings. On CT cavities were a hallmark of reactivation TB (Figure 2) [10]. In a few cases, reactivation TB presents primarily as a tuberculoma, a well-circumscribed, round or oval pulmonary lesion measuring from 0.5 to even 4 cm in diameter (Figure 3B) [10,11,12].

Miliary TB is caused by hematogenous dissemination of the bacterium and accounts for approximately 2–6% of primary TB cases. It may occur in immunocompromised patients and in reactivation forms. In reactivation TB, miliary dissemination may occur alongside classic parenchymal changes or be the sole pulmonary abnormality (Figure 3) [13]. Radiographically and on HRCT, miliary TB presents as countless 1–3 mm nodules randomly distributed across both lungs (Figure 3) [13]. Associated features often include interlobular septal thickening and a fine intralobular reticular pattern.

On 18F-FDG PET-CT scans, tuberculomas may show increased radiotracer uptake due to heightened glucose metabolism associated with active granulomatous inflammation, potentially leading to false-positive interpretations for malignancy. In these cases, 11C-choline PET-CT imaging may aid in differential diagnosis tuberculomas from malignant pulmonary lesions such as lung cancer [14,15].

Ultimately, a definitive diagnosis of TB can only be confirmed by isolating *M. tuberculosis* from a patient-derived specimen through culture. Nonetheless, diagnosing TB remains challenging, primarily due to the slow growth rate of the organism, which complicates laboratory culture procedures.

## 3. Non-Tuberculous Mycobacteria

### 3.1. Epidemiology and Pathophysiology

The non-tuberculous mycobacteria (NTM) represent a diverse group of ubiquitous, low-virulence pathogens primarily associated with infections of the cervical lymph nodes, skin, soft tissues, and lungs. Pulmonary NTM infections are increasingly prevalent, with *Mycobacterium avium* complex (MAC) and *Mycobacterium kansasii* being the most common implicated species [16].

MAC comprises multiple closely related mycobacterial species that are often indistinguishable using conventional microbiological techniques, necessitating genetic analysis for accurate identification.

*M. avium* includes four subspecies: *M. avium* subsp. *avium*, *silvaticum*, *paratuberculosis*, and *hominissuis*. Each of these subspecies is associated with different clinical burden. Pulmonary infections are predominantly attributed to subsp. *avium*, whereas the subsp. *hominissuis* is more commonly linked to gastrointestinal involvement.

MAC is the leading cause of NTM-related infections in humans, with the lungs representing the most frequently affected anatomical site [16,17,18].

MAC is a group of nonmotile, non–spore-forming, gram-positive, acid-fast bacilli, widely distributed in the environment. Infection rates are higher among women (female-to-male ratio of up to 1.6:1), possibly related to differences in pulmonary susceptibility or exposure [16].

MAC transmission occurs predominantly through inhaling infected droplets, although gastrointestinal exposure can also occur.

From a histopathological perspective, the granulomatous reaction induced by NTM is essentially indistinguishable from that caused by *M. tuberculosis*.

### 3.2. Clinical Findings

NTM infection, particularly MAC, may be asymptomatic. In some cases, patients affected by NTM infection may exhibit persistent cough (productive or dry), dyspnea, and hemoptysis—often linked to coexisting bronchiectasis and airway inflammation [17].

### 3.3. Diagnosis

Definitive diagnosis requires microbiological isolation of the organism. According to the Infectious Diseases Society of America guidelines, the evaluation of patients with suspected NTM infection should include, at minimum, radiologic, microbiologic, and clinical assessments, with particular emphasis on excluding pulmonary TB [16].

Radiologic manifestations of NTM pulmonary infections are generally not influenced by the specific mycobacterial species involved.

In immunocompetent individuals have been identified two primary radiologic patterns of disease: the upper lobe cavitary form, typically in older male with pre-existing chronic pulmonary conditions, and the nodular bronchiectatic form, in middle-aged woman without history of lung disease.

**Figure 4:**
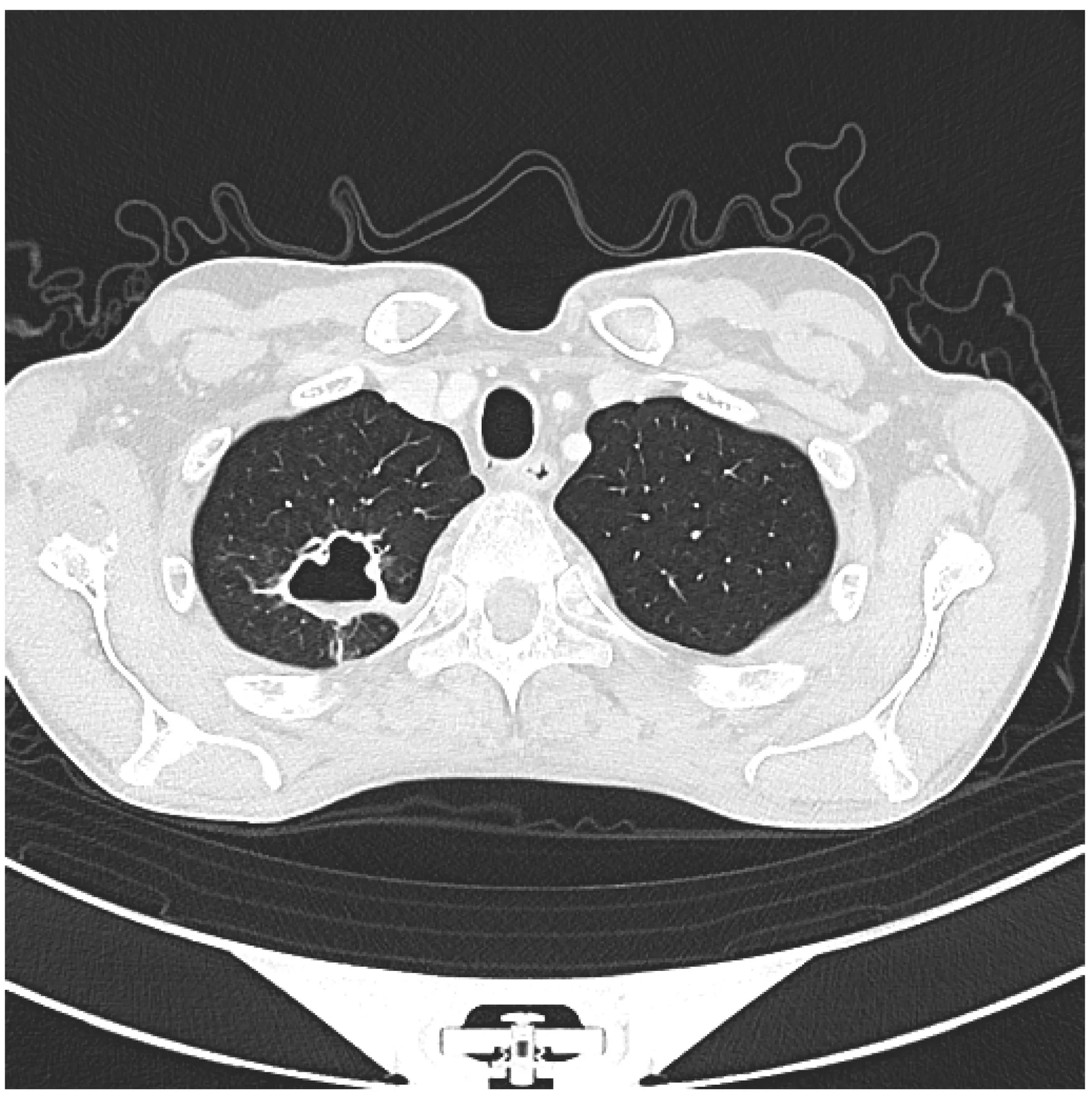
HRCT, axial plane. CT scan shows the cavitary radiologic pattern of MAC infection in a male patient. A single cavitated lesion can be noted in the right upper lobe. This lesion could be very difficult to differentiate from other type of cavitary lesions and notably from a TB lesion.

In the cavitary pattern, imaging reveals heterogeneous nodular opacities and cavitary lesions, closely resembling those of post primary TB, but with a more indolent clinical course (Figure 4).

**Figure 5:**
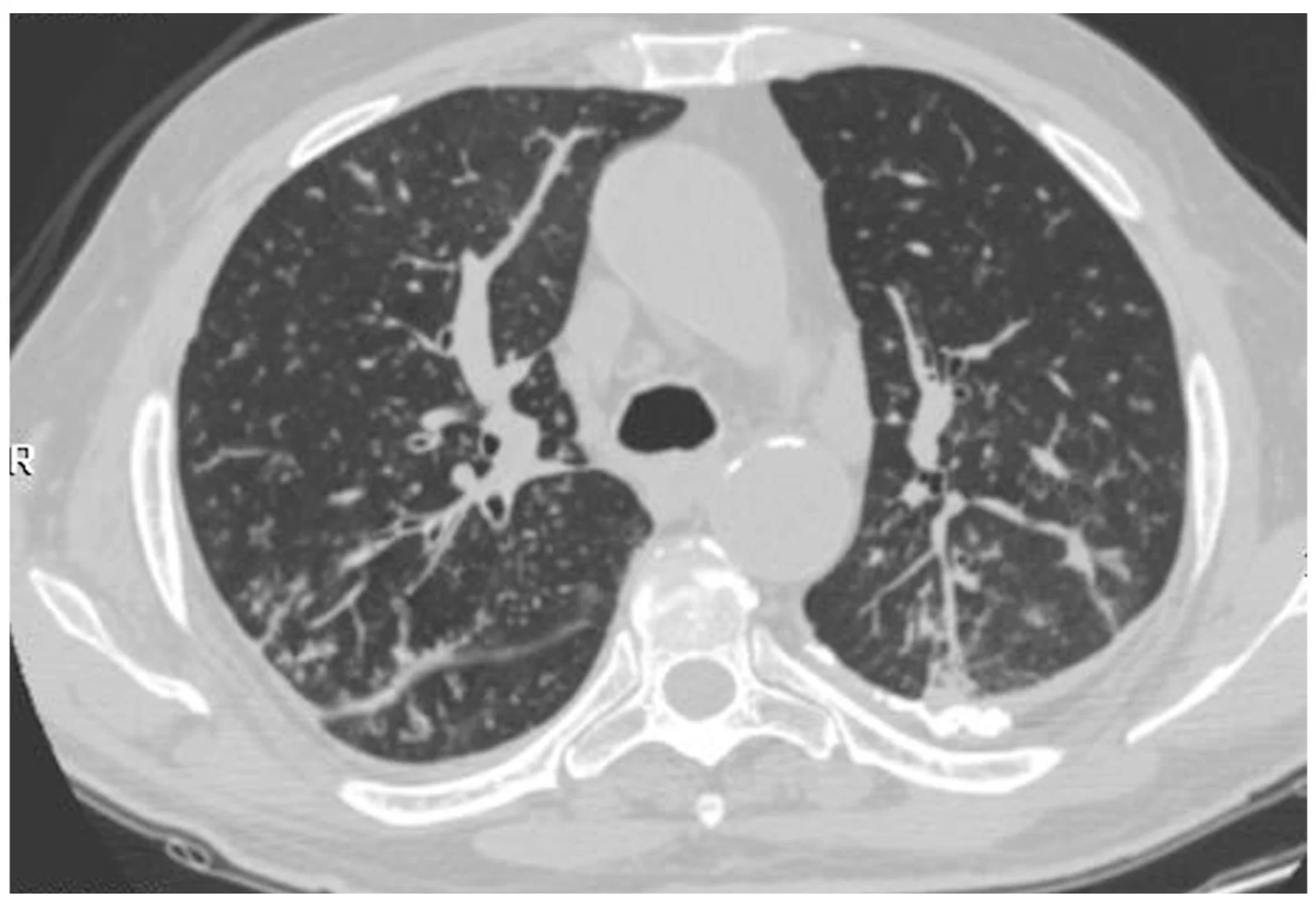
HRCT, axial plane. CT scan shows the nodular bronchiectatic form of MAC infection in a male patient, although this pattern is more frequent in female patients. In this case, there is a HRCT tree-in-bud pattern, with involvement of the distal airways and numerous centrilobular micronodules in the right upper lobe and lingula. This radiological pattern is more uncommon in the bacterial GLDs but has to be included in the differential diagnosis of other type of infectious and non-infectious bronchiolitis.

In contrast, the nodular bronchiectatic form, which is traditionally considered a display of airway colonization, is characterized by bronchiectasis and numerous branching centrilobular nodules, affecting usually the lingula and right middle lobe (Figure 5).

Histopathological evaluation from transbronchial lung biopsy specimens frequently reveals granulomatous inflammation, a sign of direct parenchymal invasion by the mycobacteria involved [19].

A significant rate of false-negative sputum cultures has been documented in individuals with this radiologic pattern, necessitating bronchoscopy or lung biopsy for definitive microbiologic diagnosis.

In immunocompromised individuals, lesions exceeding 2 cm in size and cavitary changes are observed more frequently compared to immunocompetent patients [20].

## 4. *Brucella*

### 4.1. Epidemiology and Pathophysiology

Brucellosis is a zoonotic infection transmitted to humans through contaminated animal-derived products or by direct contact with infected animals. Brucellosis is endemic in specific geographic areas, like the Middle East, Mediterranean basin, and regions of Central and South America [21].

*Brucella* consist of small aerobic intracellular coccobacilli and currently includes up to ten species, each associated with a preferred animal host: for *B. melitensis*, sheep and goats; for *B. abortus*, cattle; for *B. suis,* pigs, etc. Among these, *B. melitensis* appear to be the most pathogenic species for humans [21].

Although Europe is generally considered a low-endemic area for Brucellosis, several hotspots remain, particularly in Mediterranean countries. Within these regions, specific populations, like travelers and agricultural workers, may face increased exposure risk.

Unlike many bacterial pathogens, *Brucella* spp. does not rely on classical virulence mechanisms such as exotoxins or cytolytic enzymes; their pathogenicity is driven by a combination of specific molecular tools [22,23,24]. Notably, the intracellular cycle of *Brucella* is classically divided into three stages: (1) Incubation, an asymptomatic phase lasting from a few days to several months, (2) acute dissemination, in which the pathogen spreads systemically and start to replicate, and (3) chronic persistence, when *Brucella* adapts to the intracellular environment, leading to long-term complications or organ involvement.

Once inside macrophages, only a small fraction (<10%) of bacteria survive localizing within modified intracellular compartments known as *Brucella*-containing vacuoles, where they manipulate vesicular trafficking (by inhibiting lysosomal fusion) and initiate intracellular replication. During adaptation to the intracellular environment, *Brucella* downregulates biosynthetic pathways, enhances aminoacidic degradation, shifts to alternative energy metabolism, and modifies its respiration to cope with low oxygen availability.

Pregnant hosts are particularly affected because the pathogen shows a strong tissue tropism, with preferential colonization of placental trophoblasts, fetal lung tissue, the reticuloendothelial system, and the reproductive tract [25,26].

From a histological perspective, *Brucella* causes a granulomatous reaction characterized by a central zone of necrosis encircled by a dense rim of epithelioid histiocytes, lymphocytes, and plasma cells. Within the necrotic core, a notable polymorphonuclear cell infiltrate is often observed. Unlike caseating granulomas seen in TB, brucelloma typically lack true caseous material, reflecting a distinct pattern of tissue response [27].

### 4.2. Clinical Findings

Brucellosis can present with a broad spectrum of nonspecific symptoms, making clinical recognition challenging. Common manifestations include intermittent fever, headache, diffuse musculoskeletal pain, fatigue, anorexia, night sweats, gastrointestinal disturbances (vomiting, diarrhea, and abdominal discomfort), and in pregnant hosts, it may lead to miscarriage [28].

Musculoskeletal complications are among the most frequent, encompassing osteomyelitis, spondylodiscitis, septic arthritis, and epidural abscesses. Less frequently, the disease may involve the liver or peritoneum, presenting as hepatic abscesses, granulomatous lesions, spontaneous bacterial peritonitis. Hematologic and neurologic sequelae, such as immune thrombocytopenic purpura and Guillain-Barré syndrome, have also been reported [25].

In lung colonization, it can manifest respiratory symptoms such as cough, dyspnea, productive sputum, pleuritic chest pain, fever, crackles, and, rarely, hemoptysis [29,30].

### 4.3. Diagnosis

Brucellosis broad and nonspecific clinical presentation frequently results in misdiagnosis or delayed detection, particularly in non-endemic areas. Inadequate clinical suspicion and overlapping symptoms with other febrile conditions often postpone correct treatment [31].

Serological assays remain fundamental for diagnosis. In individuals repeatedly exposed to *Brucella* spp., these tests may produce inconclusive results because of low sensitivity and specificity. The Rose Bengal Test is commonly employed as a rapid screening method. Nevertheless, despite its high sensitivity, the test’s positive predictive value can be markedly reduced in settings of low disease prevalence, especially when serum dilution techniques are not applied.

On chest radiography, the most frequently observed abnormalities include perihilar and peribronchial infiltrates, followed by pulmonary nodules, consolidation, lung abscesses, and pleural effusion. Fine miliary mottling is the most concerning radiographic signs, because it may indicate hematogenous dissemination of the infection. Brucellosis can also be associated with bilateral hilar lymphadenopathy, and histopathological analysis of lymph nodes often reveals non-caseating granulomas, closely mimicking sarcoidosis [2,23].

On chest CT, *Brucella* infection may show solitary pulmonary nodules, ground-glass opacities (GGO) and thickening of the perivascular and peribronchial walls, and centriacinar emphysema. Pleural effusion and lymphadenopathy can also be found, occasionally presenting with central necrosis, and sometimes caseous [24,32].

On FDG PET-CT imaging *Brucella* infection is characterized by multiple foci of variably increased FDG uptake in the lungs, mediastinum, and hilar regions. These patterns are nonspecific and can be associated with both malignant and infectious or inflammatory processes. When no dominant primary lesion is detected, the possibility of an infectious etiology such as brucellosis or TB should be suspected. Confirmatory diagnosis is then guided by serological testing (e.g., *Brucella* agglutination titers) and microbiological cultures.

In differential diagnosis clinical features that favor brucellosis over TB include fever, excessive night sweats, hepatosplenomegaly, lymphadenopathy, and common musculoskeletal symptoms such as fatigue and arthralgia. Laboratory tests may reveal normal leukocyte counts, mildly elevated inflammatory markers, and increased albumin levels. Although imaging findings may overlap with other infections, they can provide supportive evidence for diagnosis.

The gold standard for diagnosis continues to be the isolation of *Brucella* spp. from bone marrow cultures. Nevertheless, because of the limited availability of resources and the prolonged time required for culture, serological testing is more commonly utilized. Despite their practical advantages, serological assays can produce false-positive results, particularly in patients with active TB, complicating the differentiation between these infections. While co-infections with *Brucella* and *M. tuberculosis* are uncommon, establishing definitive microbiological evidence for both pathogens remains a significant challenge [33].

## 5. *Coxiella burnetii*

### 5.1. Epidemiology and Pathophysiology

*Coxiella burnetii* is a pathogen taxonomically placed within the phylum Proteobacteria, alongside genera such as *Legionella*, *Francisella*, and *Rickettsiella* [34].

In humans, *C. burnetii* produces a systemic infection, acute or chronic, also defined as Q fever. Although the pathogen is globally distributed, it circulates among numerous animal species, with human involvement occurring only sporadically [34].

Of particular interest is the French Guiana strain MST17, which demonstrates a strong preference for pulmonary tissue and is considered one of the most virulent *C. burnetii* strains identified to date [34].

*C. burnetii* is a very small (0.4–1 μm in length and 0.2–0.4 μm in width), non-motile, obligate intracellular, highly pleomorphic bacterium. Although it resembles gram-negative organisms, it lacks a true capsule. Its cell envelope consists of an inner and an outer membrane. The outer membrane is composed of phospholipids, lipopolysaccharide (LPS), and a variety of protein components useful in the “phase variation,” with LPS characterizing phase I and specific proteins defining phase II [34].

This condition has a global distribution, except for a few isolated regions such as New Zealand. It primarily affects adults in midlife, with a marked predominance in males, probably due to occupational exposures and the protective role of 17-β-estradiol in women.

Most cases are reported during the spring and summer months, reflecting livestock reproductive cycles and agricultural practices. Numerous reservoirs have been identified, ranging from wildlife to domestic species, particularly goats, sheep, and cattle, as well as various tick vectors [34].

Among the possible transmission mechanisms, inhalation of contaminated droplets represents the predominant route. These aerosols are typically generated from infected animal tissues, especially placental material, as well as feces and urine.

The organism exhibits a replication cycle encompassing three morphologically distinct forms identifiable via electron microscopy: the large cell variant (LCV), the small cell variant (SCV), and the small dense cell variant (SDC). The LCV represents the metabolically active intracellular form, while the SCV and SDC are highly resilient extracellular forms capable of withstanding extreme environmental conditions and becoming aerosolized, thereby serving as the primary infectious particles.

Replication is believed to begin when SCVs are internalized by macrophages through phagocytosis and reside inside the phagolysosomes. The fusion of multiple phagolysosomes creates a single acidic vacuole. This acidic environment inhibits phagolysosomal activity and prevents host cell apoptosis, while simultaneously providing nutrients for *C. burnetii* and shielding it from antibiotics. Within this compartment, SCVs replicate via binary fission and convert into LCVs. These LCVs then undergo a “sporogenic-like” differentiation at one pole, generating the dense, compact SDC form and reducing metabolic activity [35,36].

Histologically, Q fever is characterized by non-caseating granulomas, often forming distinctive fibrin-ring (doughnut) granulomas, with a central lipid vacuole surrounded by a ring of fibrin and infiltrating macrophages, lymphocytes, and occasional giant cells. These granulomas are a hallmark of Q fever and help distinguish it from other granulomatous infections such as TB [36].

### 5.2. Clinical Findings

The clinical picture tends to be insidious, with nonspecific symptoms. Acute disease presents a wide clinical spectrum. A good proportion of infected individuals, up to 60%, experience no noticeable symptoms. Others develop a systemic illness that may resemble a viral syndrome or present as pneumonia. In approximately 4–5% of cases, the infection evolves into a localized, persistent, unfavorable process, commonly involving the heart or liver; the outcome is largely influenced by host-related factors [37].

Primary infection has considerable variability: while many patients remain asymptomatic, others progress to significant systemic disease. This heterogeneity is thought to reflect the interplay between individual characteristics, age, sex, comorbidities, and immune competence, and the infecting strain’s genotype.

When symptomatic, acute Q fever frequently begins with high fever and severe headache, with retro-orbital pain considered particularly characteristic.

The first presentation, typical of middle-aged man without previous lung disease, is atypical pneumonia, which can range between minimal radiographic abnormalities to extensive multilobar consolidation. The second presentation consist in a febrile hepatitis with elevated transaminase levels and distinctive histologic “doughnut-shaped” granulomas.

Cardiac manifestations, although less common, are highly suggestive of the diagnosis. Although endocarditis is typically associated with chronic disease, even if an autoimmune-mediated acute endocarditis has been reported by Million et al. [37]. Neurologic involvement can also occur, including meningitis, meningoencephalitis, and in rare instances, Guillain–Barré syndrome.

A portion of patients progresses to chronic infection, the most serious manifestation of which is endocarditis. Other chronic complications include infection of vascular aneurysms or prosthetic grafts. These infections often advance slowly, unrecognized until fistula formation, vertebral involvement, or psoas muscle abscess. Other chronic forms consist of persistent musculoskeletal infection: children often present with multifocal osteomyelitis, whereas adults more commonly develop infection in prosthetic joints or the spine.

Maternal infection can interfere with fetal development and may result in miscarriage, still-birth, or premature delivery, with the highest risk occurring in early gestation. In contrast, children typically experience a mild acute disease course, frequently remaining asymptomatic [37].

### 5.3. Diagnosis

Imaging plays a central role in the evaluation of Q fever, particularly in differentiating it from other infectious and non-infectious conditions. Radiologic studies, including chest radiography, HRCT, and FDG PET-CT, provide critical information on the extent, distribution, and nature of organ involvement. These modalities not only help identify pulmonary and systemic manifestations but are also essential for guiding clinical management, especially in atypical or chronic presentations.

Persistent lymphadenitis represents an uncommon manifestation of infection, yet it can closely resemble malignant processes [37].

Chest radiography lacks specificity for Q fever. It can show unilateral, single-segment opacities without a consistent lobar predilection. While Gikas et al. reported a tendency toward upper-lobe involvement, Jacobson et al. found the lower lobes more commonly affected. As a result, distinguishing Q fever pneumonia from other community-acquired pneumonias based on radiographs alone is not easy [38].

**Figure 6:**
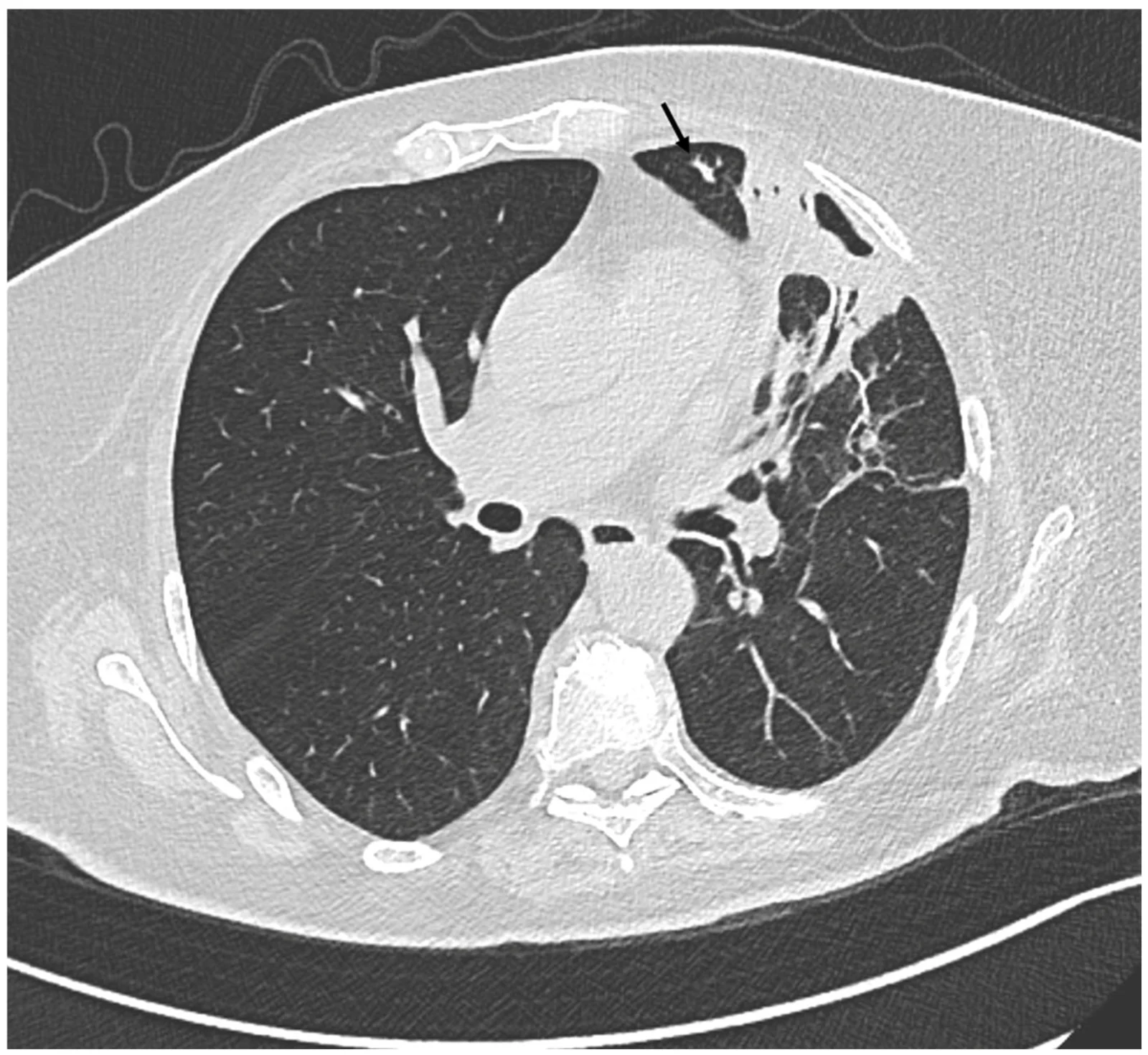
HRCT, axial plane. Case of an acute Q fever pneumonia: HRCT shows an area of airspace consolidation in the inferior lingular segment, accompanied by interstitial thickening and nonspecific parenchymal nodules (black arrow).

HRCT in acute Q fever pneumonia typically demonstrates areas of airspace consolidation or nodular opacities (Figure 6), sometimes accompanied by a surrounding ground-glass halo (halo sign) [39]. These findings may appear in a patchy, segmental, or lobar pattern frequently involving the right lower lobe. Additional findings may include interstitial edema and small, nonspecific nodules. Notably, consolidation when present tends to be non-segmental (Figure 6) [40].

Necrotizing pneumonia is likewise uncommon, although necrotizing granulomatous lesions may occur, particularly in immunocompromised individuals.

Across several studies, community-acquired pneumonia due to *C. burnetii* often appear with multilobe, bilateral consolidations in association with GGO, lymphadenopathy, and/or nodular lesions. Lymphadenitis appears to be a particularly characteristic feature of the disease, consistent with prior reports that also describe substantial mediastinal lymph node involvement in chronic Q fever [35].

According to Kouijzer et al., 18F-FDG PET-CT plays a significant diagnostic role in chronic Q fever, contributing to identification of persistent infection in 13.5% of cases even after serology and PCR testing had been completed. During follow-up, PET-CT identified ongoing focal infection in patients who present low antibody titers and no clinical symptoms. Furthermore, PET-CT findings influenced management decisions, leading to treatment modifications at the time of diagnosis and during subsequent follow-up [41].

## 6. *Francisella tularensis*

### 6.1. Epidemiology and Pathophysiology

*Francisella tularensis* is a highly virulent Gram-negative bacterium responsible for tularemia, an acute febrile zoonotic disease.

*Francisella* is the sole genus within the family *Francisellaceae* and, based on small subunit of RNA, belongs to the γ-subclass of Proteobacteria.

*F. tularensis* is further divided into five subsp.: *tularensis* (type A), *novicida*, *mediasiatica*, *holarctica* (type B), and a variant of *holarctica* found only in Japan. *F. tularensis* subsp. *tularensis* and *holarctica* are the only pathogenic for humans [41,42].

Tularemia is endemic across much of the Northern Hemisphere, yet has not been observed in the Southern Hemisphere, except for an isolate of subsp. *novicida* in Australia, which did not cause typical tularemia. Infection can occur via the skin, conjunctiva, oral ingestion, or respiratory tract. After a short incubation period (3–5 up to 14 days), patients generally experience flu-like symptoms. The clinical course then varies depending on the route of infection, potentially progressing to one of six classical forms [40].

From a histological perspective, tularemia is characterized by necrotizing granulomas, with central necrosis that may resemble caseous necrosis, surrounded by epithelioid histiocytes and occasional neutrophilic infiltrates. These granulomas can closely mimic those seen in TB, making microbiological confirmation essential for accurate diagnosis [42].

### 6.2. Clinical Findings

Human infection typically occurs through contact with wild animals, particularly hares and small rodents, arthropod bites (primarily Ixodidae ticks and mosquitoes), or exposure to contaminated environmental sources [43].

Clinical manifestations of tularemia vary deeply according to the route of infection, as ulceroglandular, oculoglandular, oropharyngeal, typhoidal, and pneumonic tularemia.

In respiratory tularemia, the classic route starts after inhalation of aerosolized bacteria and may have a wide spectrum of symptoms, from mild systemic manifestations to severe pneumonia. Type A strains are particularly virulent, producing high fever, chills, dyspnea, cough, chest pain, mental alterations, and pulse temperature dissociation (mimicking typhoid disease). Radiographic appearances are variable and may mimic pneumococcal pneumonia, TB, or even malignancy. Type B infections are generally more indolent, although can present significant pulmonary involvement [43].

### 6.3. Diagnosis

Diagnosis of tularemia is often delayed due to the mild and non-specific initial symptoms, and low clinical suspicion. Isolation of *F. tularensis* from clinical specimens is challenging, achieved in fewer than 10% of cases. Blood cultures may yield the organism in cases of bacteremia, while less commonly it can be isolated from a swab taken from the affected area or from biological fluids [40].

Radiological evaluation of pulmonary tularemia starts with chest radiography showing opacities and hilar lymphadenopathy [44].

**Figure 7:**
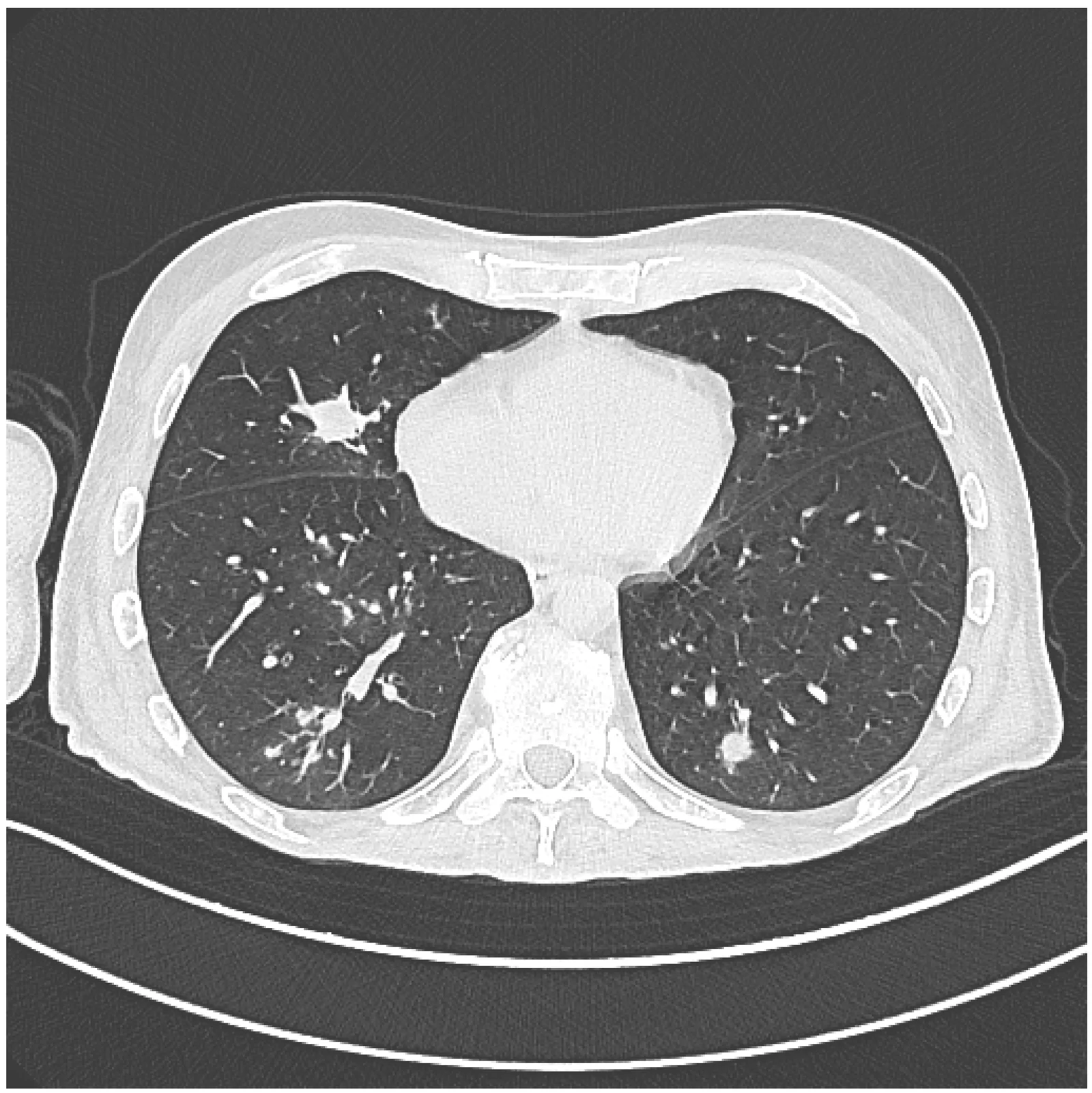
HRCT, axial plane. HRCT scans reveal multiple bilateral nodular opacities, prevalent in peripheral regions. Pulmonary tularemia in this case mimic neoplastic lesions, but in the right lower lobe the findings are more indicative of an infectious disease.

CT scans reveal a dense lobar consolidation or nodular opacities and lymphadenopathy, usually in peripheral regions (Figure 7). When present, a distinguishing feature of the disease is the presence of central necrosis within both lymph nodes and pulmonary lesions [45].

Pulmonary tularemia may mimic neoplastic lesions or community-acquired pneumonia, particularly in immunocompromised individuals.

Pulmonary complications include pneumonia, lung abscesses, and acute respiratory distress syndrome (ARDS). Rare cases of myocarditis linked to *F. tularensis* have also been reported in endemic areas.

In pediatric patients, tularemia often presents more pronounced cervical lymphadenopathy. The infection can also manifest unexpectedly in immunocompromised individuals, including those with neutrophil deficiencies, transplant-related immunosuppression, malignancies, HIV infection, or implanted medical devices, even in the absence of a clear exposure.

PET-CT can also be performed often showing uptake patterns that can mimic lung cancer in over 50% of cases [45].

As the isolation of *F. tularensis* remains uncommon and often incidental, serology remains the cornerstone of tularemia diagnosis, although often must wait two weeks. Consequently, early-stage patients presenting with localized symptoms (pharyngitis or conjunctivitis) or systemic forms (pneumonic or typhoidal) may be seronegative [45].

## 7. *Bartonella henselae*

### 7.1. Epidemiology and Pathophysiology

*Bartonella henselae* present a persistent bacteremia across diverse reservoir species and are responsible for a wide range of human diseases. The most widely recognized clinical manifestation is cat scratch disease (CSD). Another manifestation is trench fever, characterized by recurrent fever, severe headache, and bone pain.

*Bartonella* spp. are facultative intracellular, Gram-negative bacilli characterized by slow growth requirements. To date, at least 22 species have been identified, of which 12 are recognized as pathogenic to humans. Within their natural reservoir hosts, *Bartonella* organisms establish chronic infection, ultimately persisting within erythrocytes [46,47].

The global incidence of CSD has been estimated at 6.4 cases per 100,000 adults and 9.4 cases per 100,000 children aged 5–9 years. Trench fever has caused both epidemic and sporadic outbreaks since the early twentieth century, especially during the World Wars. Nowadays, homeless populations represent the group at greatest risk for trench fever [48].

Members of the genus *Bartonella* have been detected in a broad spectrum of mammalian hosts and has numerous arthropod vectors. Numerous virulence determinants have been identified that facilitate host-cell invasion, persistence within erythrocytes, angiogenic responses, and evasion of immune surveillance [48].

The histopathologic manifestations of human *Bartonella* infection are heterogeneous and include focal granulomatous inflammation in CSD, characterized by granuloma formation with central necrosis and the recruitment of histiocytes, lymphocytes, and multinucleated giant cells, often accompanied by prominent neoangiogenesis and endothelial proliferation within and around the granuloma, and multi-focal angioproliferative lesions in bacillary angiomatosis (BA), intravascular proliferation in endocarditis, and a proposed immune-mediated inflammatory response in meningoencephalitis without direct bacterial invasion. BA is more frequent in immunocompromised patients. In CSD, lymph node specimens typically show patchy necrotizing granulomatous inflammation with stellate abscesses and variable leukocytic infiltration. In contrast, BA is characterized by proliferation of small blood vessels lined by enlarged, plump endothelial cells, accompanied by leukocyte infiltration and focal necrosis but lacking granuloma formation, a pattern like that observed in systemic bartonellosis [49].

### 7.2. Clinical Findings

The clinical manifestations of *Bartonella* infection in humans vary widely depending on both the infecting species and the immune status of the host.

CSD usually begins with a localized cutaneous lesion at the site of bacterial inoculation, most commonly following a cat scratch or bite. The initial lesion evolves through vesicular, erythematous, and papular stages and typically appears within 7–10 days after exposure. This is followed by regional lymphadenopathy, while systemic manifestations occur less frequently.

Pulmonary involvement, though uncommon, may present as an infectious pneumonia characterized by dyspnea, productive cough, fever, fatigue, and hypoxemia. In severe cases, necrotizing pneumonia may develop, with cavitation, liquefactive necrosis, and extensive parenchymal destruction [47].

*B. quintana* causes trench fever, characterized by relapsing or persistent fever, malaise, headache, bone pain, and splenomegaly. In contrast, *B. bacilliformis* cause a biphasic disease, with an initial febrile hemolytic phase (Oroya fever) followed weeks later by eruptive cutaneous lesions known as verruga peruana.

### 7.3. Diagnosis

Imaging plays a pivotal role in raising suspicion of *Bartonella* infection, particularly in patients with pets (e.g., cats).

On plain chest radiography, pulmonary bartonellosis manifests as a large area of consolidation, most often involving the right upper lobe, with mediastinal widening or bulging [48].

**Figure 8:**
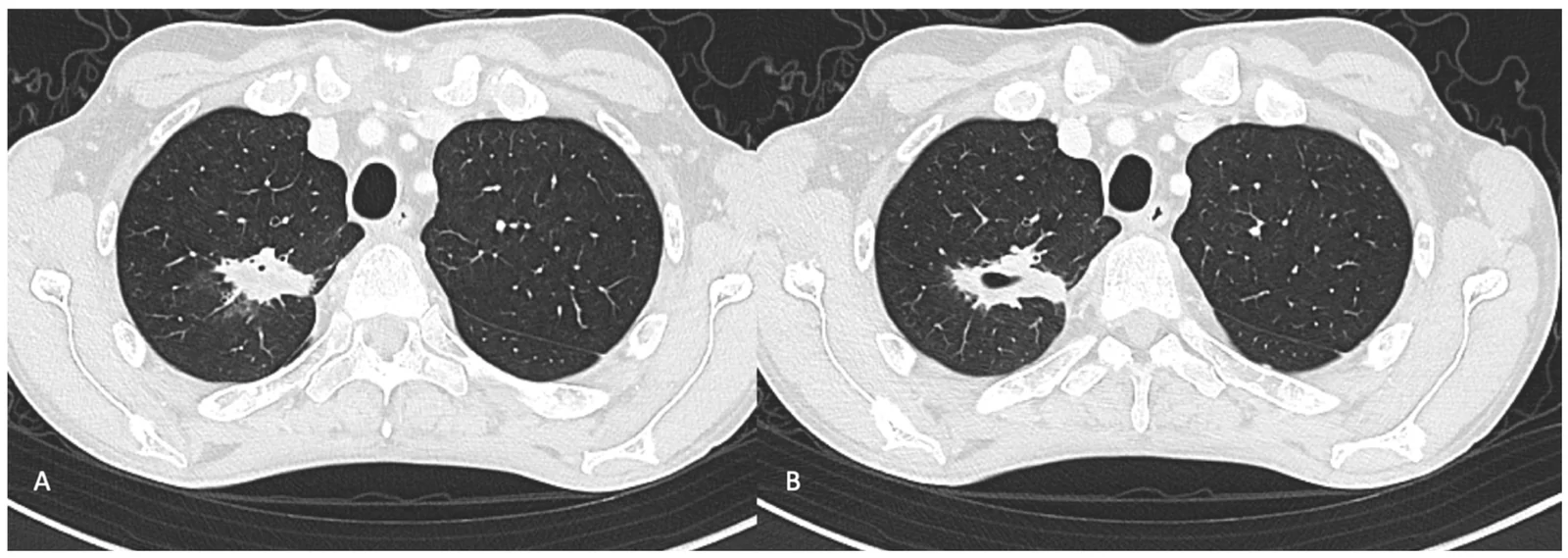
HRCT, axial plane. In this case, the patient had a strong clinical suspicion of bartonellosis and was therefore examined with HRCT rather than CECT. In the first CT scan (**A**) it appears as a large mass in the right upper lobe. At the one-month follow-up after specific therapy (**B**), unfortunately, the lesion had cavitated, but it did not evolve into a necrotizing form, so intralesional cavitation was detected but no findings indicative of necrosis—as multiple internal air–fluid levels surrounded by areas of parenchymal consolidation. Without clinical and laboratory data, HRCT findings may be very similar to TB or MAC infection, making radiological diagnosis very challenging.

On CECT, pulmonary bartonellosis usually appears as a large enhancing mass, most common in the upper lobes or with hilar distribution (Figure 8A). Mediastinal and hilar lymph nodes are often associated, mimicking a malignant process [47].

When the infection evolves into necrotizing pneumonia, CT reveals thick-walled cavitary lesions containing multiple internal air–fluid levels and surrounded by areas of parenchymal consolidation (Figure 8B). With disease progression, further complications can develop, more frequently as bronchopleural fistula formation.

Pleural involvement is common, often with large, sometimes loculated, pleural effusions or, in severe infections, empyematous collections [49].

The ulterior diagnostic workup of *Bartonella* infection relies on a combination of serology, culture, histopathology, and molecular techniques. Although *Bartonella* can be isolated in culture, this method is rarely used because the bacteria grow very slowly (requiring several week) and need specialized culture conditions (optimal culture requires direct inoculation of samples onto solid media supplemented with hemin, followed by incubation under hypercapnic conditions). For these reasons, culture is generally not recommended for standard clinical diagnosis. Serologic testing, therefore, represents the primary diagnostic approach in most patients.

## 8. *Actinomyces*

### 8.1. Epidemiology and Pathophysiology

Thoracic actinomycosis is most frequently observed in alcohol abusers or in patients with seizure disorders. Rarely, infection may also result from a human bite or following surgical procedures in the oral cavity, particularly in patients with poor oral hygiene [50].

*Actinomyces* spp. are filamentous, Gram-positive bacilli and are part of the normal human commensal microbiota, predominantly colonizing the oropharynx, gastrointestinal tract, and urogenital tract, where *Actinomyces* are nonvirulent [50].

More than 30 *Actinomyces* species have been identified, among these, *Actinomyces israelii* is the most isolated from human infections [51].

The incidence of actinomycosis is higher in males between 20 to 60 years old (M:F ratio is 3:1) and a greater prevalence has been reported in populations with low socioeconomic status [52].

Pathogenicity typically requires the presence of companion bacteria that facilitate evasion of host immune defenses and disruption of mucosal barriers.

Following breach of the mucosal surfaces and establishment of infection, the host mounts a marked inflammatory response characterized by both suppurative and granulomatous features. In fact, histologically, it is featured by a chronic, suppurative granulomatous inflammatory response with non-caseating granulomas and abundant neutrophilic infiltrates [53].

### 8.2. Clinical Findings

The most common infection site is the cervicofacial region, causing “lumpy jaw syndrome”. Pulmonary involvement, instead, occur primarily from aspiration of oropharyngeal or gastrointestinal secretions.

Pulmonary actinomycosis may manifest with acute or subacute symptoms, although most cases are recognized in the chronic stage. Initial clinical features are vague and include low-grade fever, fatigue, and weight loss, followed over time by a persistent productive cough, hemoptysis, dyspnea, and thoracic pain. Progressive parenchymal involvement frequently results in cavitary lesions and the development of sinus tracts, a feature regarded as highly characteristic of this infection. In advanced disease, especially among immunocompromised patients, significant hypoxemia and severe respiratory compromise may develop, potentially progressing to ARDS [50].

Chronic lung diseases associated with tissue destruction and impairment of normal mucosal defenses, such as chronic obstructive pulmonary disease, bronchiectasis, and TB also confer increased susceptibility. *Actinomyces* may extend directly to the mediastinum and pleura from this site.

Given the propensity of *Actinomyces* spp. to invade devitalized tissue, individuals with parenchymal destruction and bronchiectasis secondary to prior TB or other pulmonary infections are particularly predisposed to secondary actinomycosis [53].

### 8.3. Diagnosis

Early and accurate diagnosis is essential to prevent the disease morbidity. Misdiagnosis is common, especially as a malignancy [54].

HRCT represents the imaging modality of choice. In cases of initial pulmonary actinomycosis, the most frequent finding is a small defined peripheral lung nodule, with or without associated interlobular septal thickening, with a slight predilection for the upper lobes. If not treated, the lesion progressively enlarges, evolving into segmental airspace consolidation, suggestive of bronchogenic spread. Over time, central areas of low attenuation may develop, with cavitary lesions that can be single or multiple, variable in size, and occasionally demonstrate rim-like peripheral enhancement on CECT.

**Figure 9:**
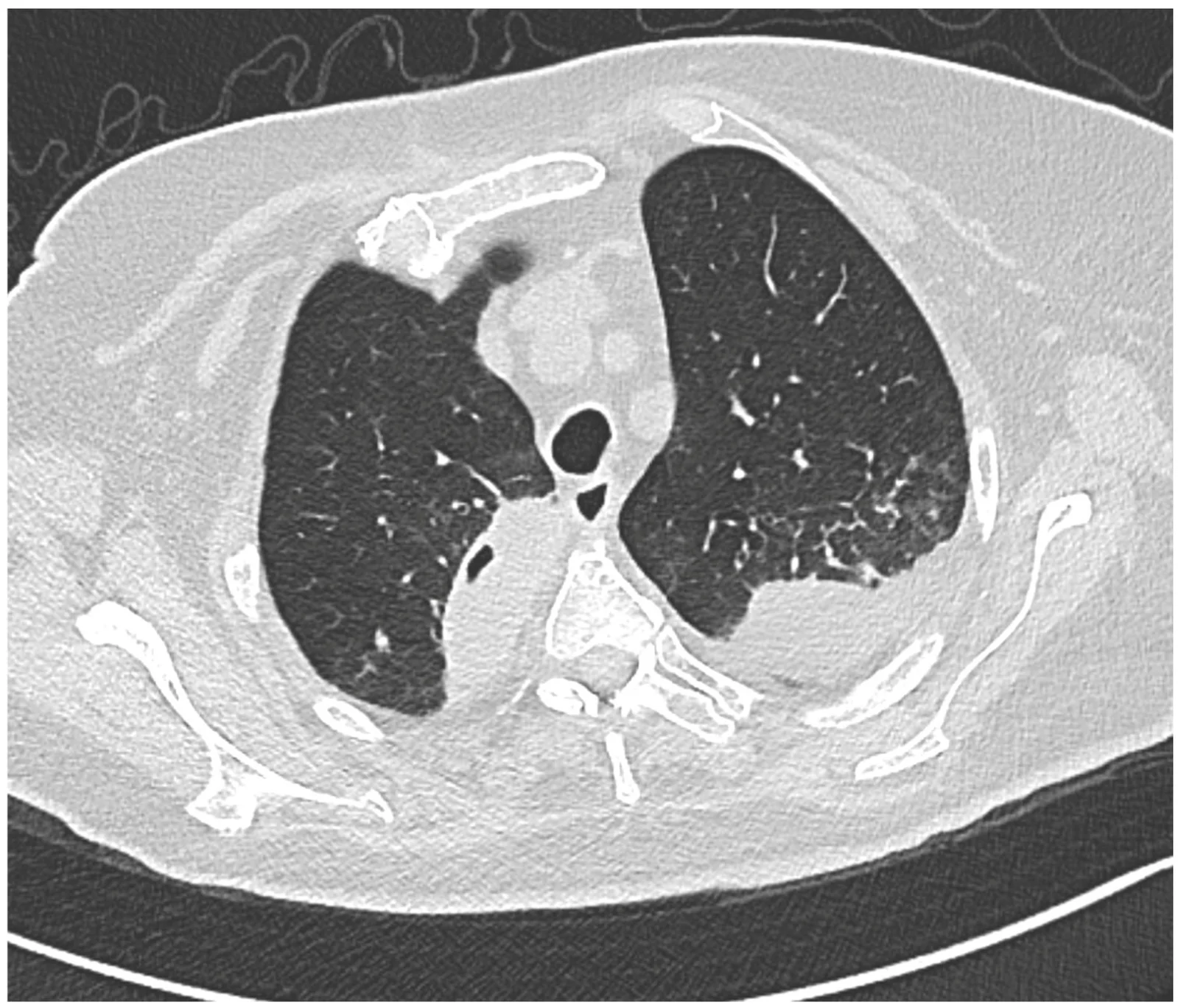
HRCT, axial plane. This patient was affected by bilateral pulmonary *Nocardia* infection. HRCT shows mass-like consolidations in the upper lobes; in the right lung the air crescent sign can be observed while in the left lung are associated interlobular septal thickening and pleural effusion. The air crescent sign makes the radiological differential diagnosis with *Aspergillus* pulmonary infection very difficult.

In advanced stages, the disease may present as a mass-like lesion with central necrotic low attenuation (Figure 9) or as extensive parenchymal destruction with extension across interlobar fissures (“transfissural spread”) or into extrapulmonary structures such as the pleura or chest wall, often frequently leading to abscess formation. Mediastinal involvement, however, is exceedingly rare. Associated imaging findings include interlobular septal thickening, bronchiectasis, mediastinal lymphadenopathy, adjacent pleural thickening, pleural effusion, and empyema [55,56].

The “air crescent sign” may also be observed, although it is not specific (Figure 9).

The diagnostic gold standard remains histopathological examination and bacterial culture of tissue obtained via biopsy. Samples may be acquired through bronchoscopy, CT-guided percutaneous biopsy, or VATS. Anaerobic cultures of pleural fluid rarely yield the organism, and sputum cultures are generally unhelpful unless cavitary disease is present [56].

On PET-CT, intense radiotracer uptake can be evident, with reported SUV ranging from 5.4 to 13.7, mimicking malignancy [57].

## 9. *Nocardia*

### 9.1. Epidemiology and Pathophysiology

*Nocardia* spp. most commonly may cause pulmonary nocardiosis, consisting of almost 73–77% of *Nocardia* infectious manifestations [58].

Infection results from inhalation of airborne *Nocardia* organisms from environmental sources (dust, etc.). Once established, the infection can remain localized or disseminate via hematogenous routes or by direct tissue extension. Immunosuppressed patients are at significantly higher risk for severe and disseminated forms [59].

*Nocardia* genus consist of a group of aerobic, filamentous, Gram-positive actinomycetes and are widely distributed in the environment (soil and organic matter) [59].

An annual incidence up to 0.87 cases per 100,000 individuals has been reported [60]. Histologically, pulmonary nocardiosis show a necrotizing, suppurative granulomatous inflammatory response, with areas of tissue necrosis with associated granulo-histiocytic infiltrates and branching filamentous Gram-positive organisms that stain weak acid fast within necrotic and inflammatory foci inside [60].

### 9.2. Clinical Findings

Clinical manifestations of *Nocardia* infection vary depending on the bacteria route, with the lungs and skin being the most frequently affected regions. Pulmonary nocardiosis represents the classic presentation, particularly in immunocompromised patients. However, cutaneous infections may also occur in immunocompetent individuals, often following direct inoculation after skin graze [58].

### 9.3. Diagnosis

Imaging plays a pivotal role in the evaluation of pulmonary nocardiosis.

On chest X-ray, *Nocardia* infection can generate a wide range of thoracic abnormalities. The most frequent consist in parenchymal consolidation, solitary and irregular or multiple lesions, involving extensive lung regions. Cavitation is considered a characteristic feature [61]. Pleural effusions may be unilateral or bilateral.

A rapid radiographic progression is common with the development of prominent nodules, masses, or dense consolidations with indistinct margins. These findings are prevalent in immunocompromised patients, including those with systemic disease and undergoing anti-inflammatory therapies [58].

**Figure 10:**
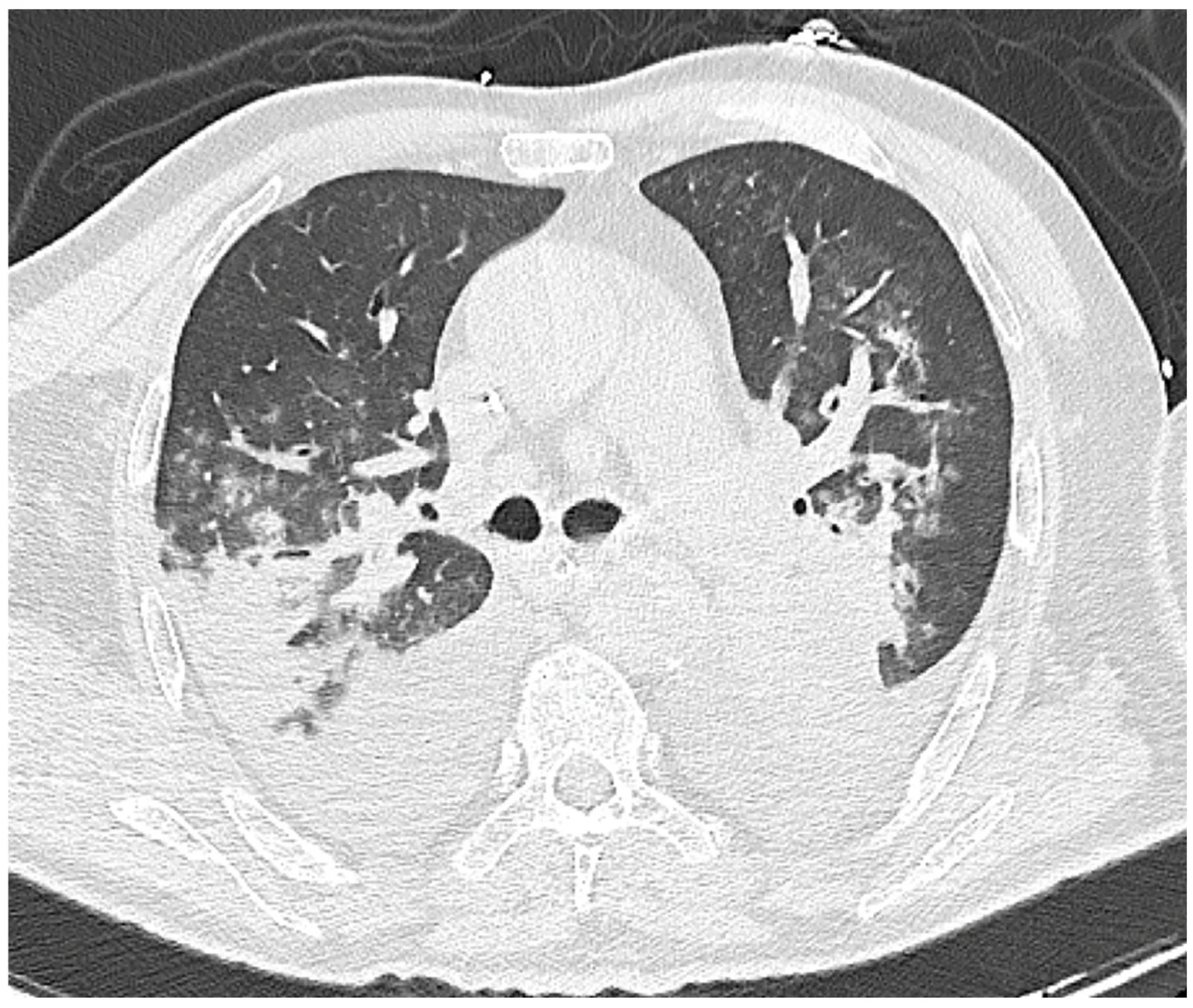
HRCT, axial plane. CT scans show an acute and advanced case of *Nocardia* lung infection, in presence of multilobar consolidations, GGO, and bilateral pleural effusion.

On CT, the most typical abnormalities include lobar or multilobar consolidation, followed in frequency by GGO and centrilobular nodules (Figure 10). In a little group of patients could be see interlobular septal thickening and reticular interstitial opacities reflecting interstitial involvement [62].

Even in this infection the “halo sign” may be identified. Mucoid impaction and bronchiectasis are frequently encountered, whereas lymphadenopathy is uncommon.

Pleural involvement, with possible evolution into empyema, can be observed in CT, typically occurring through direct extension of adjacent parenchymal disease. In chronic inflammatory settings, involvement can lead to pleural thickening.

As in pulmonary TB and actinomycosis, nocardial infection can extend into the chest wall, resulting in abscess formation or phlegmon (empyema necessitans). CECT is essential for detecting this spread. Features suggesting chest wall involvement include fluid collections adjacent to affected lung and pleura, as well as muscular edema with loss of normal fat planes and peri-muscular fat stranding.

The differential diagnosis for pulmonary nocardiosis includes other lung cavitation disease, such as alternative infections, vasculitis, and malignancies. Actinomycosis and TB are the most usual CT differential diagnoses with *Nocardia*; however, actinomycosis typically does not disseminate to the central nervous system and is more commonly associated with poor dentition and aspiration of infected oropharyngeal secretions. TB, instead, shares a lot of risk factors, but the absence of lymphadenopathy is more suggestive of nocardiosis [62].

## 10. Other Bacterial Pathogens

GLDs may be the result of various types of bacterial infections, especially in immunocompromised patients.

In addition to the bacteria listed above, which most commonly cause pulmonary granulomas, rare cases of granulomas caused by other less frequent pathogens have been reported. For example, Zudekoff et al. reported a case of identified *Cutibacterium acnes* as the etiologic agent in chronic pleural disease. Chest X-ray revealed bilateral pleural effusions, confirmed on CT that show rounded atelectasis. Lung biopsies demonstrated chronic pleural inflammation with fibrinous pleuritis and lung non-necrotizing granulomas [63].

In rare cases, especially in secondary syphilis caused by Treponema pallidum, a granulomatous reaction with non-caseating granuloma can be noted, composed of aggregates of epithelioid histiocytes within a lymphoplasmacytic infiltrate, and multinucleated giant cells are occasionally present [64].

*Pseudomonas andersonii*, a recently described Gram-negative bacillus, has been implicated in chronic pulmonary granulomatous disease. Although its reservoir remains unknown, *P. andersonii* is frequently isolated alongside other microorganisms, including MAC or fungi, yet the pulmonary lesions consistently demonstrate granulomatous and cavitary lesion, indicating its role in chronic necrotizing lung infections [65]. In addition, *Pseudomonas aeruginosa* can also cause cavitary lung lesions that, although less common, are clinically very significant, representing necrotizing granulomatous inflammation with localized tissue destruction. They appear as low-attenuation, air-filled spaces within consolidation or ground-glass areas, consistent with liquefactive necrosis. Peripheral and upper-lobe predominance may indicate regions of impaired ventilation, ischemia, or altered host defense, conditions favoring bacterial persistence and granuloma formation [66].

## 11. Conclusions

Infectious GLDs consist of a variable spectrum of pulmonary diseases featured by the presence of lung granulomatous lesions caused by microbial agents.

The diagnostic approach to GLDs could be very challenging and relies on integrated evaluation of clinical history, laboratory investigations, and imaging studies.

Imaging is fundamental for the detection and characterization of lung granulomas, and HRCT is considered the gold standard technique for their assessment. Notably, HRCT can evaluate the number, size, and distribution of the lung granulomas and may suggest an infectious etiology based on specific imaging patterns. CECT and PET-CT have a fundamental role in the study of the activity and the metabolic evaluation of the granulomatous lesions. Despite the high diagnostic performance of HRCT, CECT, and PET-CT in detecting and following granulomatous lung lesions, accurate differential diagnosis remains challenging. This difficulty arises from the wide variety of underlying pathogenic mechanisms and the substantial overlap in imaging appearances among different GLDs.

The aim of this review is to describe the spectrum of infectious GLDs caused by bacterial pathogens, highlighting their different patterns in order to assist radiologists in recognizing these entities and improving diagnostic accuracy.

## Figures and Tables

**Table 1:** The table provides a summary of all the main findings and helps to outline the key differences between the infectious GLDs examined.

	Distribution	Cavitation	Granuloma	Lymph Nodes	HRTC Pattern	Key Findings
*Mycobacterium tuberculosis*	Upper lobe (apical- posterior segment)	Present, thick-walled	Caseous necrosis	Present (especially children)	Patchy consolidation, nodules, cavitation	Cavitations Tree in bud Miliary
Non-Tuberculous Mycobacteria	Upper Lobe (cavitary form); Middle lobe/lingula (nodulary form)	Present	Caseous necrosis	Present (especially children)	Bronchiectasis, centrilobular nodules, cavitation	Cavitations Tree in bud
*Brucella*	Variable, usually Peribroncho-vascular	Rare	Non Caseous necrosis	Present	Nodules, GGO	Miliary, mimicking sarcoidosis
*Coxiella burnetii*	Segmental, Lower Lobe (DX>SN)	Rare	Doughnut (fibrin ring)	Present	Consolidation, nodules, GGO	Halo sign
*Francisella tularensis*	Peripheral	Present	Necrotizing	Present	Consolidation, nodules	Central necrosis lesions and lymph nodes
*Bartonella henselae*	Upper lobe, Hylar	Present	Necrotizing	Present	Mass-like consolidation	Tumor-like lesion
*Actinomyces*	Peripheral, upper lobe	Present	Suppurative	Rare	Consolidation evolving into mass	Transfissural spread
*Nocardia*	Lobar/Multilobar	Present	Necrotizing/ suppurative	Rare	Nodules, consolidation, GGO	Halo sign, empyema necessitans

## Data Availability

No new data were created or analyzed in this study. Data sharing is not applicable to this article.
